# Soybean-Derived Phytoalexins Improve Cognitive Function through Activation of Nrf2/HO-1 Signaling Pathway

**DOI:** 10.3390/ijms19010268

**Published:** 2018-01-16

**Authors:** Ji Yeon Seo, Bo Ram Kim, Jisun Oh, Jong-Sang Kim

**Affiliations:** School of Food Science and Biotechnology (BK21plus Program), Kyungpook National University, Daegu 41566, Korea; quftkfka@gmail.com (J.Y.S.); brkim8595@naver.com (B.R.K.); j.oh@knu.ac.kr (J.O.)

**Keywords:** soybean, phytoalexins, glyceollins, neuroprotection, MAPK, Nrf2, HO-1

## Abstract

As soy-derived glyceollins are known to induce antioxidant enzymes in various types of cells and tissues, we hypothesized that the compounds could protect neurons from damage due to reactive oxygen species (ROS). In order to examine the neuroprotective effect of glyceollins, primary cortical neurons collected from mice and mouse hippocampal HT22 cells were challenged with glutamate. Glyceollins attenuated glutamate-induced cytotoxicity in primary cortical neuron isolated from mice carrying wild-type nuclear factor (erythroid-derived 2)-like 2 (Nrf2), but the compounds were ineffective in those isolated from Nrf2 knockout mice, suggesting the involvement of the Nrf2 signaling pathway in glyceollin-mediated neuroprotection. Furthermore, the inhibition of heme oxygenase-1 (HO-1), a major downstream enzyme of Nrf2, abolished the suppressive effect of glyceollins against glutamate-induced ROS production and cytotoxicity, confirming that activation of HO-1 by glyceollins is responsible for the neuroprotection. To examine whether glyceollins also improve cognitive ability, mice pretreated with glyceollins were challenged with scopolamine and subjected to behavioral tests. Glyceollins attenuated scopolamine-induced cognitive impairment of mice, but failed to enhance memory in Nrf2 knockout mice, suggesting that the memory-enhancing effect is also mediated by the Nrf2 signaling pathway. Overall, glyceollins showed neuroprotection against glutamate-induced damage, and attenuated scopolamine-induced memory deficits in an Nrf2-dependent manner.

## 1. Introduction

Reactive oxygen species (ROS) are involved in a number of neurodegenerative diseases including Alzheimer’s disease, Parkinson’s disease, and Huntington’s disease [1]. Therefore, natural antioxidants are expected to prevent or mitigate the progression of these disorders. Our previous studies have demonstrated that glyceollins, a family of phytoalexins derived from soybeans, exhibited strong antioxidant activity, both in vitro and in vivo [2,3]. In particular, the compounds were found to exert antioxidant effects through activation of the nuclear factor (erythroid-derived 2)-like 2 (Nrf2)/Kelch-like ECH-associated protein 1 (Keap1) signaling pathway [3,4]. Under physiological conditions, Nrf2, which is a master regulator of the expression of various antioxidant enzymes, is present as a dimeric form with Keap1, and is subjected to ubiquitin-dependent proteosomal degradation [5,6]. However, upon exposure to electrophiles or oxidative radicals, it is separated from Keap1 and translocates into the nucleus to act as a transcriptional activator of numerous proteins including antioxidant enzymes. Heme oxygenase 1 (HO-1), one of the major downstream enzymes to Nrf2 transcription factor, is involved in catalyzing heme into carbon monoxide, free Fe^2+^, and biliverdin, which is converted to bilirubin, a potent intracellular antioxidant. In the normal brain, the expression of HO-2 is constitutive, abundant, and fairly ubiquitous, whereas HO-1 is limited to small populations of scattered neurons and neuroglia. In contrast, the *ho-1* gene is extremely sensitive to induction by a wide range of pro-oxidant and other stressors [7]. Natural HO-1 inducers have drawn much attention as they mediate protection from brain aging and neurodegeneration, mainly though enhancement of antioxidant potential [8]. As activation of Nrf2-mediated antioxidant enzymes including HO-1 protects neurons from oxidative damage [9,10,11,12], we hypothesized that glyceollins could attenuate ROS-induced neuronal damage and cognitive impairment. Thus, the objective of this study is to examine the neuroprotective and cognition-enhancing effects of glyceollins.

## 2. Results

### 2.1. Effect of Glyceollins on Glutamate-Induced Cytotoxicity in Primary Cortical Neurons Isolated from Nrf2 Wild-Type and Knockout Mice

**Figure 1:**
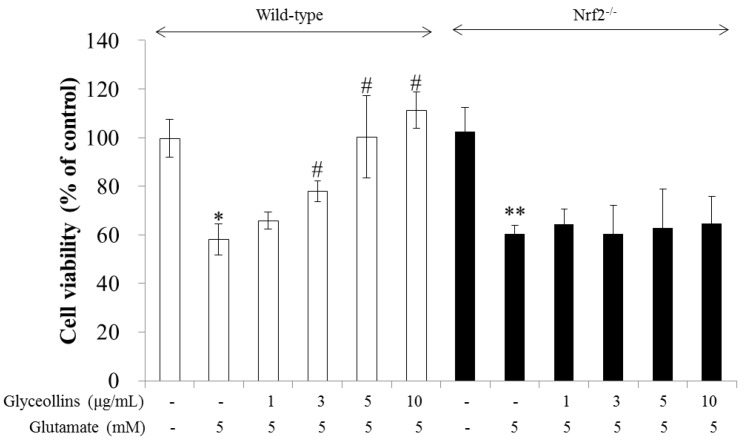
Attenuation of glutamate-induced excitotoxicity by glyceollins in primary cortical neurons. Cortical neurons isolated from fetal brain of C57BL/6 wild-type mice (white bars) or Nrf2 knockout (black bars) mice were treated with 1–10 μg/mL of glyceollins in the presence of glutamate for 24 h and followed by MTT assay. Bars represent mean ± standard deviation (SD, *n* = 4). *, **, Statistically significant difference from the untreated control (*p* < 0.05). #, Statistically significant difference from the positive control group treated with glutamate alone (*p* < 0.05).

Glyceollins suppressed glutamate-induced excitotoxicity in primary cortical neurons isolated from the fetal brains of Nrf2 wild-type C57BL/6 mice in a concentration-dependent manner in the range of 1–10 μg/mL, as represented by the white bars in Figure 1. In contrast, the protective effects of glyceollins were not observed in the primary cortical neurons isolated from the fetal brains of Nrf2 knockout C57BL/6 mice, as represented by the black bars in Figure 1.

### 2.2. Effect of Mitogen-Activated Protein Kinase (MAPK), PI3K, Nrf2, and HO-1 on the Attenuation of Glutamate-Induced Oxytosis by Glyceollins

**Figure 2:**
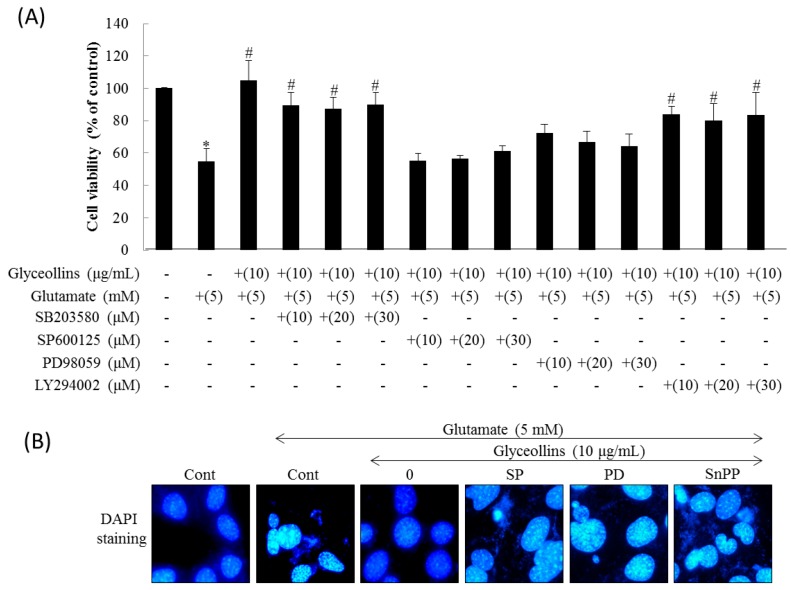
Effects of inhibitors for MAPK, PI3K, and HO-1 on the attenuation of glutamate-induced neurotoxicity by glyceollins. Mouse hippocampal HT22 cells were seeded at a density of 2 × 10^3^ cells/well into 96-well plate and 3 × 10^4^ cells/well into the gelatin-coated 24-well plate for assessment of morphology or DAPI staining, respectively. (**A**) HT22 cells were treated with MAPK or PI3K inhibitors in the presence of glyceollins (10 μg/mL) and glutamate (5 mM) for 24 h, followed assessing cell viability by MTT assay. Bars represent mean ± SD (*n* = 4). Marks above each bar indicated the significant difference. *, Significantly different from the untreated control (*p* < 0.05). #, Significantly different from the positive control group treated with glutamate alone (*p* < 0.05); (**B**) HT22 cells were treated with ERK inhibitor (SP), JNK inhibitor (PD), or HO-1 inhibitor (SnPP) in the presence of glyceollins (10 μg/mL) and glutamate (5 mM) for 24 h, followed by DAPI staining and visualizing under fluorescent microscope to evaluate cytotoxicity (magnification, 400×).

The inhibition of proliferation of HT22 cells induced by glutamate was restored to the control level by 10 μg/mL of glyceollins. The cytoprotective effect of glyceollins against glutamate was nullified by co-treatment with an extracellular signal-regulated kinase (ERK) inhibitor (10–30 μM SP600125), or a c-Jun N-terminal kinase (JNK) inhibitor (10–30 μM PD98059) (Figure 2A). However, a p38 inhibitor (10–30 μM SB203580) and a phosphatidylinositol 3 kinase (PI3K) inhibitor (10–30 μM LY294002) did not attenuate the protective effect of glyceollins from glutamate-induced cell death (Figure 2A).

When treated with 5 mM glutamate for 24 h, HT22 cells showed morphology indicative of apoptosis, including cellular shrinkage, DNA condensation, and decreased cell volume. Glyceollins reversed the morphological changes caused by glutamate (5 mM). Furthermore, the suppression of apoptotic change of cells by glyceollins was not diminished by the co-treatment with an ERK inhibitor (10–30 μM SP600125), a JNK inhibitor (10–30 μM PD98059) or a HO-1 inhibitor (40 μM SnPP) (Figure 2B).

### 2.3. Suppression of Intracellular ROS (Reactive Oxygen Species) Level by Glyceollins

**Figure 3:**
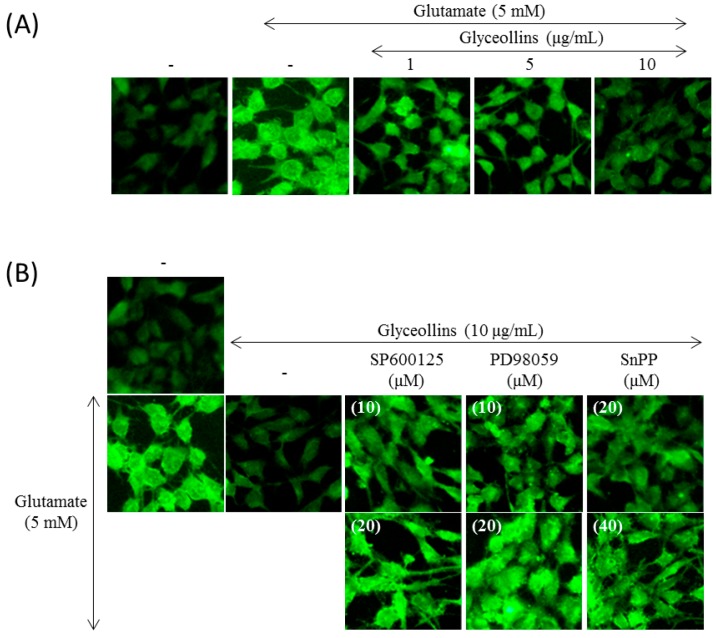
Effects of inhibitors for MAPK, PI3K, and HO-1 on the suppression of glutamate-induced intracellular ROS production by glyceollins. (**A**) HT22 cells were treated with various concentrations of glyceollins for 8 h, followed by visualizing cell image under fluorescent microscope after staining with a DCFDA dye to assess intracellular ROS levels (magnification, 400×); (**B**) Cells were pretreated with inhibitors for 2 h and subsequently incubated in the presence of glutamate and glyceollins for another 8 h. Cell images were visualized by fluorescence microscopy after staining with a DCFDA dye.

Exposure of HT22 cells to glutamate (5 mM) for 8 h resulted in an approximately 50% increase in ROS production compared to the untreated control. However, the increased level of intracellular ROS caused by glutamate insult was lowered by pre-exposure of the cells to glyceollins (10 μg/mL) (Figure 3A).

### 2.4. Effect of MAPK and HO-1 Inhibitors on Glyceollins-Mediated Suppression of ROS Production

While glyceollins mitigated the glutamate-induced increase in intracellular ROS level, their effect was abolished by MAPK and HO-1 inhibitors. More specifically, suppression of intracellular ROS production by glyceollins was abrogated by co-treatment with inhibitors for any of the following: ERK1/2 (10–30 μM SP600125), JNK (10–30 μM PD98059), HO-1 (40 μM SnPP) as shown in Figure 3B. 

### 2.5. Stimulation of Nuclear Translocation of Nrf2 and HO-1 Expression by Glyceollins 

**Figure 4:**
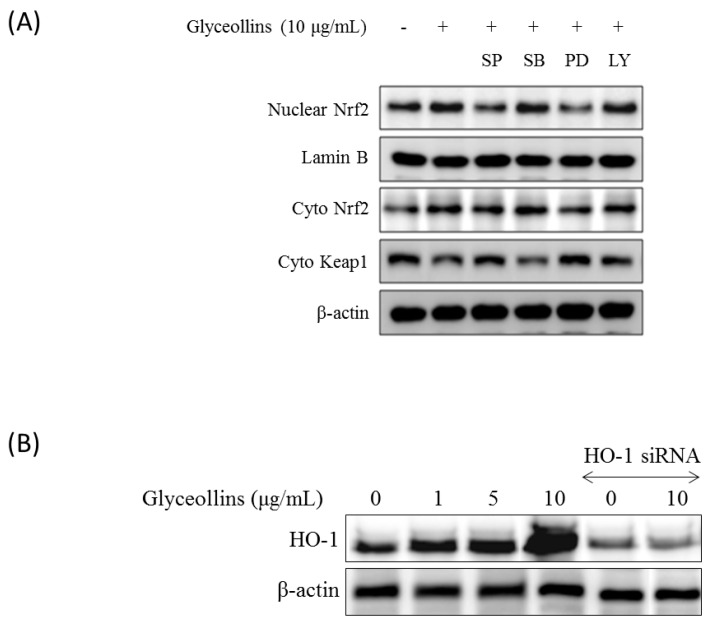
Glyceollin-induced nuclear localization of Nrf2 and HO-1 expression. (**A**) HT22 cells were incubated with glyceollins (10 μM) and MAPK and PI3K inhibitors for 6 h and separated into nuclear and cytoplasm extracts. Each extract was used for Western blot analysis. Lamin B and β-actin were used as a protein loading control. Inhibitors used are as follows; SP (ERK inhibitor, SP600125), SB (a p38 inhibitor, SB203580), PD (JNK inhibitor, PD98059), or LY (PI3K inhibitor); (**B**) SH-SY5Y cells were incubated with various concentrations of glyceollins for 12 h, followed by Western blot analysis for HO-1 expression. In addition, SHSY5Y cells were transiently transfected with HO-1 siRNA and subsequently treated with glyceollins for 24 h. Whole cell extract was collected and used for Western blot analysis of HO-1 expression. All experiments were conducted in duplicate.

Cytosolic and nuclear levels of Nrf2 were significantly enhanced in HT22 cells by a 6-h treatment with glyceollins (Figure 4A). The expression of *Hmox1*, which is a gene downstream of the Nrf2 signaling pathway, was also induced by glyceollins in a dose-dependent manner (Figure 4B). However, co-treatment with MAPK inhibitors abolished the stimulatory effect of glyceollins on nuclear translocation of Nrf2 (Figure 4A). 

### 2.6. Effect of MAPK Inhibitors on ARE-Mediated Transcriptional Activation by Glyceollins

**Figure 5:**
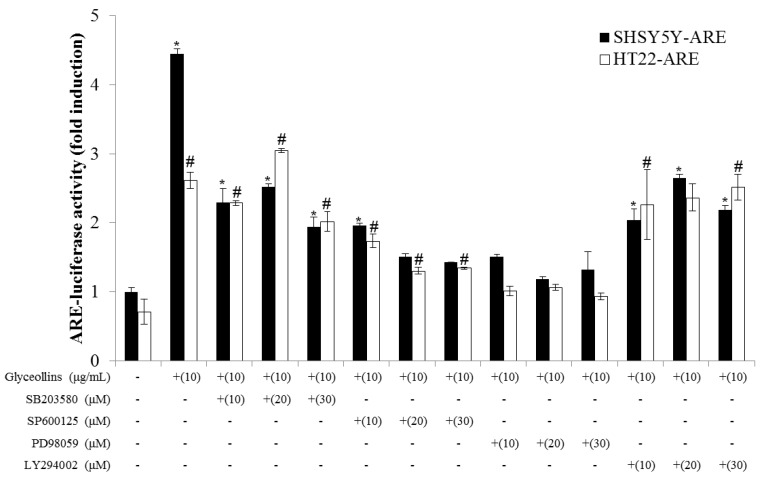
Effect of inhibitors of MAPK, PI3K on Nrf2 transcriptional activation by glyceollins, as evaluated by reporter assay. SHSY5Y-ARE or HT22-ARE cell lines were treated with glyceollins in the presence or absence of inhibitors for MAPK and PI3K for 16 h followed by measuring luciferase activity. Bars represent mean ± SD (*n* = 4). *, Significantly different from the untreated control (*p* < 0.05) in SHSY5Y-ARE cells. #, Significantly different from the untreated control (*p* < 0.05) in HT22-ARE cells.

To confirm that activation of the Nrf2 signaling pathway is mediated through the interaction of nuclear Nrf2 with antioxidant response element (ARE), the *cis*-elements in the promoter region of antioxidant enzyme genes, transfectant SHSY5Y-ARE, and HT22-ARE cells containing reporter ARE-luciferase gene were exposed to various concentrations of glyceollins for 16 h. Reporter luciferase activity was dramatically increased by glyceollins, whereas ARE-luciferase induction by glyceollins was suppressed by ERK or JNK inhibitors, particularly in the HT22-ARE cell line (Figure 5), consistent with the cytotoxicity data shown in Figure 2. 

### 2.7. Improvement of Cognitive Function by Glyceollin Treatment of Scopolamine-Induced Mouse Model of Amnesis

**Figure 6:**
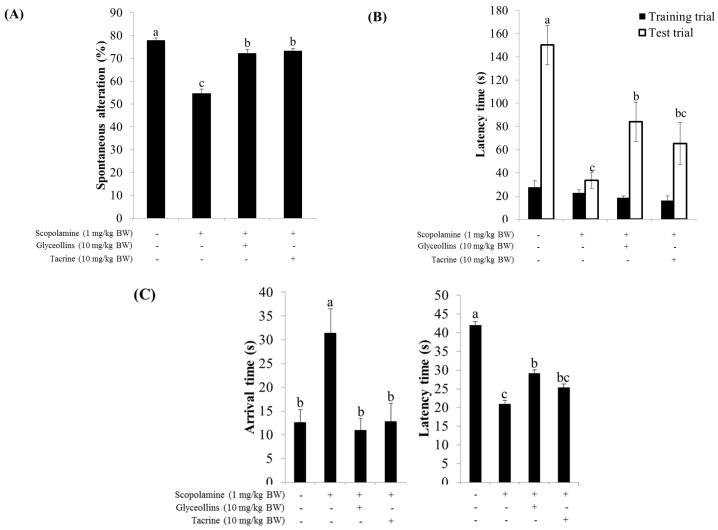
Effect of glyceollins on cognitive function in Nrf2 wild-type mice. Nrf2 wild-type C57BL/6 mice (five weeks old, *n* = 9–15) were pretreated with glyceollins (10 mg/kg bw) 24 h before cognitive function or behavior tests, and then subjected to Y-maze (**A**); passive avoidance (**B**); and Morris Water maze (**C**) tests after injected *i.p*. with scopolamine (1 mg/kg bw). Values are mean ± SD (*n* = 9–15). Different alphabetical letters (a, b, c) indicate statistically significant differences.

Y-maze spontaneous alternation, which measures the willingness of rodents to explore new environments, was impaired by scopolamine but partially restored by co-administration with glyceollins or tacrine, a well-known acetylcholine esterase (AChE) inhibitor (Figure 6A).

A passive avoidance test showed that oral administration of glyceollins (10 mg/kg) significantly restored the decreased latency induced by scopolamine (1 mg/kg) in mice. This beneficial effect of glyceollins was comparable to that of tacrine (Figure 6B). 

A Morris water maze task showed that spatial learning and memory, measured by time spent on or near the platform, was shortened by scopolamine and partially restored by glyceollins but not by tacrine, and the delay in time to reach the platform due to scopolamine injection was significantly improved by the administration of glyceollins (Figure 6C). 

**Figure 7:**
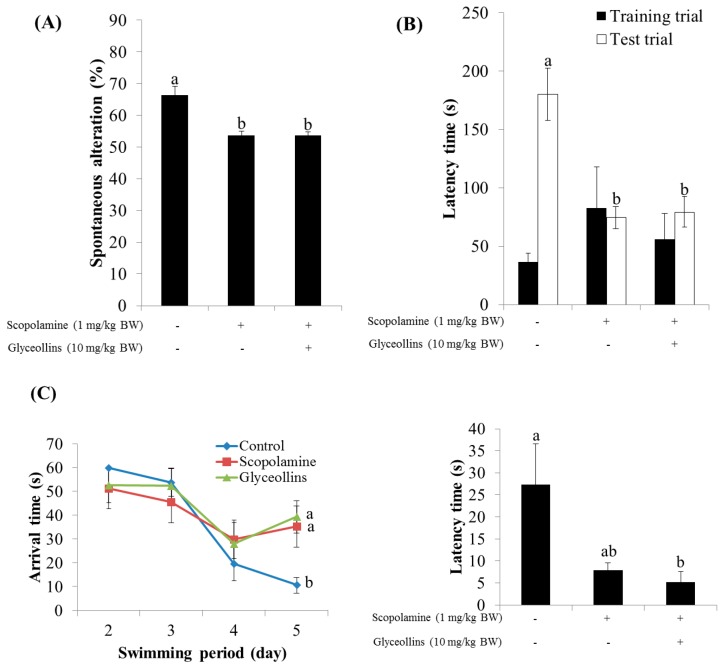
Effect of glyceollins on cognitive function in Nrf2 knockout mice. Nrf2 knockout C57BL/6 mice (five weeks old, *n* = 7) were pretreated with glyceollins (10 mg/kg bw) 24 h before cognitive function or behavior tests, and then subjected to Y-maze (**A**); passive avoidance (**B**); and Morris Water maze (**C**) tests after injected *i.p*. with scopolamine (1 mg/kg bw). Different alphabetical letters (a, b) on bars indicate statistically significant differences.

**Figure 8:**
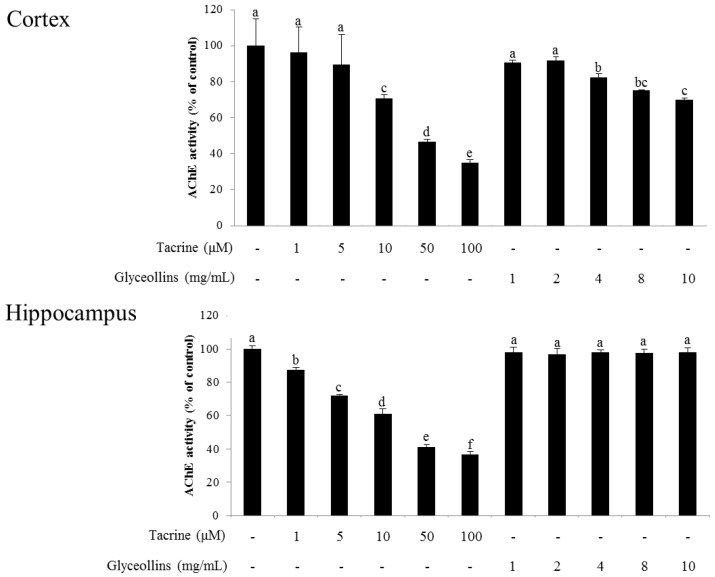
Inhibitory activity of glyceollins against AChE in mouse brain cortical and hippocampal homogenates. Neuronal cells isolated from either cortical or hippocampal regions were homogenated and used for assaying the inhibitory activity of glyceollins against AChE. Values are mean ± SD (*n* = 7). Different alphabetical letters (a–f) on bars indicate significant differences. Cortical and hippocampal neuron cells were isolated from the mouse brain and their homogenates were used for assaying the inhibitory activity of glyceollins against AChE.

Interestingly, the ameliorative effect of glyceollins in the associative or spatial learning and memory-impaired mouse model was not observed in Nrf2 knockout mice (Figure 7). Furthermore, glyceollins exhibited an inhibitory effect on AChE activity in neurons isolated from the cortex at high concentrations of glyceollins but not in cells from the hippocampus (Figure 8).

## 3. Discussion

Antioxidant enzymes whose expression is regulated by the Nrf2 signaling pathway have been reported to exert a neuroprotective effect, with the potential to prevent ROS-associated neurodegenerative disorders [13]. Among antioxidant enzymes, HO-1 attracted much attention due to its ability to produce a potent endogenous antioxidant bilirubin. The knockout of the *ho-1 gene* led to high susceptibility to oxidative insult in mice, emphasizing the significant role of HO-1 as a cellular antioxidant. Furthermore, cognitive impairment caused by oxidative stress is prevented by antioxidants such as vitamin E [14]. In the current study, we found that glyceollins, activators of the Nrf2/HO-1 signaling pathway, protect primary cortical neurons from glutamate-induced cytotoxicity in an Nrf2/HO-1 dependent manner. More specifically, the neuroprotective effect of glyceollins was observed in the primary cortical neurons isolated from Nrf2 wild-type mice but not in those from Nrf2 knockout mice (Figure 1). Furthermore, neuroprotective effect of glyceollins appears to be mediated by its ability to scavenge glutamate-induced intracellular ROS, which, in turn, is attributable to the activation of the Nrf2/HO-1 signaling pathway (Figure 3 and Figure 4). This notion was further confirmed by the findings that the inhibitors of MAPK such as ERK and JNK abolished the cytoprotective effect of glyceollins in mouse hippocampal HT 22 cells. The MAPKs are known to be upstream regulators of the Nrf2 signaling pathway (Figure 2 and Figure 5) [15,16]. 

Furthermore, glyceollins were found to prevent mnemonic and cognitive deficits induced by scopolamine in a mouse model of amnesia. Impairment of learning and memory in mice exposed to scopolamine was improved by glyceollins, as measured by the Y-maze, passive avoidance, and Morris water maze tests. Interestingly, the beneficial effect of glyceollins on cognitive function was not observed in Nrf2 knockout mice, suggesting the potential association of Nrf2 with cognitive function. Scopolamine, a nonselective muscarinic antagonist, has frequently been employed to clinically inhibit the effects of parasympathetic activation and has been used as a reference drug to induce age- and dementia-related cognitive deficits in neuropharmacological studies on healthy humans and animals [17,18]. While the compound induces short-term memory impairment by interfering with the binding of acetylcholine with the muscarinic receptor M1, M2, and M3, a high dose of scopolamine (e.g., 0.1 mg/kg or higher) has also been speculated to exert systemic effects such as neuronal damage by increasing ROS production in the brain, leading to working-memory deficits. This notion is consistent with our findings that the impairment of exploratory behavior and cognitive function of mice induced by scopolamine, as measured by Y-maze and Morris water maze tests, respectively, was not reversed by glyceollins in Nrf2 knockout mice [19].

Interestingly, glyceollins also inhibited AChE activity at a concentration of 4 mg/mL or higher, in a dose-dependent manner. Therefore, the attenuation of scopolamine-induced cognitive deficit by glyceollins could be mediated through activation of the Nrf2 signaling pathway as well as the inhibition of AChE. However, glyceollins failed to inhibit AChE activity in the hippocampus responsible for short-term memory, suggesting that they exert an anti-amnesic effect by inducing Nrf2/antioxidant enzymes signaling pathway rather than inhibiting AChE [19]. 

Our previous studies demonstrated that glyceollins produced in raw soybeans and exposed to various kinds of stress, including fungal infection and ultraviolet irradiation, induce antioxidant enzymes such as HO-1 and NAD(P)H:quinone oxidoreductase in an Nrf2-dependent manner and exert direct antioxidant activity [2,3]. In this study, we found that glyceollins exert neuroprotective effects by upregulating heme oxygenase-1 expression through the activation of the Nrf2 signaling pathway. However, it is not clear how HO-1 protects neurons from oxidative stress. A recent study indicates that carbon monoxide, one of the enzymatic products of HO-1, is more likely to be responsible for the improvement of cognitive performance than bilirubin or biliverdin, as cognitive skills were improved by the CO-donor tricarbonyldichlororuthenium (II) but not by biliverdin [20]. In addition, further study is required to confirm that the neuroprotective effect of glyceollins is fully responsible for the restoration of cognitive impairment caused by scopolamine insult.

## 4. Materials and Methods

### 4.1. Reagents and Chemicals

All cell culture media were obtained from Welgene (Daegu, Korea). Neurobasal medium, fetal bovine serum (FBS), N-2 supplement, and B-27 supplement were obtained from Gibco (GIBCO BRD, Gaithersburg, MD, USA). Most of the chemicals used in this study were obtained from Sigma (St. Louis, MO, USA). Antibodies were purchased from Santa Cruz Biotechnology (Santa Cruz, CA, USA).

### 4.2. Preparation of Glyceollins

Glyceollins were kindly provided by Soon Sung Lim, Hallym University (Chuncheon, Kangwon-do). Glyceollins I, II, and III were prepared according to the method described previously [21]. Briefly, the glyceollin-rich extract was prepared by inoculation of soybean seed with *Aspergillus sojae* in a dark chamber, at 25 °C, for three days. After homogenization in 80% aqueous ethanol and incubation, the filtered extract was freeze-dried. The ethyl acetate-soluble fraction of the crude extract was subjected to silica gel column chromatography. One fraction was subjected to thin-layer chromatography and liquid chromatography–mass spectrometry to confirm the existence of glyceollins, after which the fraction was evaporated under reduced pressure.

### 4.3. Cell Culture

The glutamate-sensitive murine hippocampal neuronal HT22 and human neuroblastoma SHSY5Y cell lines were kindly provided by Dr. Dong-Seok Lee, College of Natural Sciences and Dr. Kyung-Sik Song, College of Pharmacy Kyungpook National University (Daegu, South Korea), respectively. Cells were grown in Dulbecco’s modified Eagle’s medium (DMEM) supplemented with 10% FBS and 100 units/mL penicillin and 100 μg/mL streptomycin at 37 °C under 5% CO_2_. Cells were subcultured at a 1:8 ratio every two days.

### 4.4. Isolation of Cortical Neurons

Primary cortical neuron cells were isolated from the fetal brains of Nrf2 wild-type C57BL/6 and Nrf2 knockout mice at a gestational age of 15 days. More specifically, the cortex was dissected and kept in ice-cold phosphate-buffered saline. The cortical tissue was dissociated to single cells by passing through a 70-μm nylon cell strainer (SPL Life Sciences, Pocheon, Korea). The cell suspension was centrifuged at 1100× *g* for 3 min, and the resulting pellets were resuspended in the plating medium (DMEM/F12, supplemented with N2 supplement, nonessential amino acid (5 mL/L), HEPES4-(2-hydroxyethyl)-1-piperazineethanesulfonic acid (3.9 g/L), sodium bicarbonate (3.7 g/L), and sodium pyruvate (55 mg/L), pH 7.2–7.4). The cells were seeded on plates coated with poly-l-lysine at a density of 3 × 10^5^ cells /well in a CO_2_ incubator (5% CO_2_/95% air, 37 °C). After cell attachment, the medium was changed into neurobasal medium containing a B-27 supplement, and supplemented with 0.5 mM glutamate. Every 48 h, cells were fed with fresh neurobasal medium. All assays were performed after one week when the neurons became mature.

### 4.5. Oxytosis Assay

Oxytosis assay was performed using previously published methods, with some modifications [22]. HT22 cells or primary cortical neurons were seeded onto 48-well plates with DMEM containing 10% FBS at an initial density of 2 × 10^3^ cells/well and incubated in 5% CO_2_ at 37 °C for 24 h or one week, respectively. Thereafter, the medium was removed and replaced by DMEM containing various concentrations of glyceollins in the presence of 5 mM glutamate. In a separate experiment, the cells were treated with glutamate and glyceollins in the absence and presence of HO-1 inhibitor (40 μM of SnPP), ERK inhibitor (10–30 μM SP600125), JNK inhibitor (10–30 μM PD98059), p38 inhibitor (10–30 μM SB203580), and PI3K inhibitor (10–30 μM LY294002). After 24 h, cultures were terminated by removing the medium from the wells and adding 3-[4,5-dimethylthiazol-2-yl]-2,5-diphenyltetrazolium bromide solution (0.25 mg/mL in culture medium). After 2 h, the solution was removed, and 200 μL of dimethylsulfoxide (DMSO) were added. After 10 min, the absorbance was determined at 570 nm using a microplate reader (Sunrise TECAN, Salzburg, Austria). The cell proliferation of each group was calculated as the absorbance of the treated group relative to the control.

A HO-1 inhibitor (40 μM of SnPP) or an ERK inhibitor (SP600125) or a JNK inhibitor (PD98059) (Figure 2A). However, a p38 inhibitor (SB203580) and a PI3K inhibitor (10–30 μM of LY294002).

### 4.6. DAPI Staining

HT22 cells were seeded in a 24-well plate, containing sterile coverslips, at a density of 3 × 10^4^ cells/well [22]. After 24 h of incubation, cells were exposed to glyceollins and glutamate for another 24 h. Cells were washed with TBS, fixed with 3.7% paraformaldehyde, and blocked with 1% BSA dissolved in TBS. The cells were then treated with 4′,6-diamidino-2-phenylindole (DAPI, 100 ng/mL). After washing with TBS twice, coverslips were mounted with a drop of mounting medium (Dako A/S, Glostrup, Denmark) and observed under a fluorescence microscope (Eclipse 80i, Nikon, Tokyo, Japan) at 400× magnification.

### 4.7. Establishment of Antioxidant Response Element (ARE) Construct Transfected Stable Cell Lines

HT22 or SH-SY5Y cells were transfected with 100 ng of pGL4.37[luc2P/ARE/Hyg] vector (Promega Corp., Madison, WI, USA) per well in a 6-well plate using Lipofectamine 2000 (Invitrogen, Carlsbad, CA, USA) according to the manufacturer’s protocol. The cells transfected with ARE element were selected by culturing in the presence of 0.2 mM hygromycin. After subculturing five times, the stable cell lines were established. Both HT22-ARE and SHSY5Y-ARE cell lines were subcultured in the presence of 0.2 mM hygromycin at a 1:5 ratio every two days.

### 4.8. Antioxidant Response Element (ARE)–Reporter Gene Assay

HT22-ARE or SHSY5Y-ARE cells were seeded in 12-well plates, with DMEM containing 10% FBS, at an initial density of 1 × 10^4^ cells/well, and incubated in 5% CO_2_, at 37 °C, for 12 h. Then medium was then replaced by serum-free DMEM, containing various concentrations of glyceollins, in the absence or presence of MAPK inhibitors. After incubation for 16 h, cells were harvested, and the luciferase activity was measured according to the protocol provided by the manufacturer (Promega Corp., Madison, WI, USA) [20].

### 4.9. Determination of Intracellular ROS Level

The intracellular level of ROS was quantified using 2,7-dichlorofluorescein diacetate (DCFDA), according to the protocol described previously [21]. Cells were seeded in a black-bottom 96-well plate (Nunc, Rochester, NY, USA) and maintained for 24 h using DMEM containing 10% FBS. Cells were further incubated in the absence or presence of sample for another 12 h. Then cells were incubated with DCFDA dissolved in DMSO (final concentration: 10 μM), for 30 min. Fluorescence was measured at 535 nm, in a microplate reader (Infinite 200, Salzburg, Austria). Most of the steps, including incubation of the reaction mixture containing dye and oxidant, washing, and fluorometric determination, were performed in the dark. The fluorescence intensity of samples was expressed as a percentage of that of the negative control, in which neither sample nor glutamate was added.

### 4.10. Western Blot Analysis

The cytoplasmic and nuclear levels of HO-1 and Nrf2 were measured using western blotting, in which proteins (2–50 μg) were separated by electrophoresis on a 12% sodium dodecyl sulfate (SDS) polyacrylamide gel (1.5 M Trizma base, 10% SDS, 30% acrylamide, 10% ammonium sulfate, tetramethylethylenediamine), at 200 V, and transferred to polyvinylidene fluoride membranes (Millipore, Bedford, MA, USA) for 70 min, at 100 V [3,10]. The membranes were blocked for at least 2 h in 1% bovine serum albumin (BSA)-Tris-buffered saline/Tween20 (TBS/T, 20 mM Tris-HCl, pH 7.4, 150 mM NaCl, 0.1% Tween20), and were incubated overnight with the antibodies anti-HO-1, anti-Nrf2, anti-Lamin B, or anti-β-actin, diluted 1:1000, at 4 °C, followed by three washes in TBS/T for 10 min each, and then incubated with horseradish peroxidase (HRP)-linked anti-mouse IgG, HRP-linked anti-rabbit IgG, or HRP-linked anti-goat IgG, for 4 h. After three 10-min washes in TBS/T, the protein bands were detected using a Super Signal^®^ West Pico Chemiluminescent Substrate (Thermo Scientific, Rockford, IL, USA). The densitometric analysis of the protein bands was performed using MultiGauge 3.0 software (Fujifilm, Tokyo, Japan).

### 4.11. Animal Behavioral Tests

Male C57BL/6J mice (four weeks old) were obtained from Daehan Biolink (Seoul, Korea), and C57BL/6J/Nrf2 knockout (*Nrf2*^−/−^) mice were kindly provided by Masayuki Yamamoto, Tohuku University, Japan. Mice were allowed a week for adaptation. The protocol for this study was approved by the Animal Care and Use Committee of Kyungpook National University (Daegu, Korea; permission number: KNU-2012-72) on 29 June, 2012. 

The cognitive function of mice was evaluated using passive avoidance, Y-maze, and Morris water maze tests, as described elsewhere [23,24,25,26]. Wild-type or Nrf2 knockout C57BL/6J male mice (five weeks old) were divided into 10 groups, with eight mice per group. Glyceollins or tacrine were orally administered. Memory impairment was induced in mice with scopolamine (1 mg/kg, intraperitoneally injected) 30 min after treatment with glyceollins or tacrine. The control group received 10% Tween 80 solution only.

### 4.12. AChE Activity Assay

The AChE assay was carried out according to Ellman’s method with some modifications [27]. Cortical and hippocampal tissues were collected separately from mice after the sacrifice. The tissues were homogenized on ice with 10 mL of phosphate buffer (12.5 mM phosphate buffer, pH 7.0 containing 400 mM NaCl) and then centrifuged at 1000× *g* for 10 min at 4 °C. The supernatant was used as the source of enzyme for the assay. Five μL of glyceollins or tacrine, 100 μL of 100 mM sodium phosphate buffer, 100 μL of the enzyme source, and 25 μL of Ellman’s reagent (10 mM 5,5′-dithio-bis (2-nitrobenzoic acid) and 15 mM sodium bicarbonate) were mixed at 25 °C for 30 min. The mixture then incubated with 10 μL of 75 mM acetylthiocholine iodide for 10 min. The absorbance was measured at 415 nm using a microplate reader (Sunrise TECAN).

### 4.13. Statistical Analysis

Data were tested by analysis of variance, followed by Duncan’s multiple range test to compare mean values of each other, using SPSS Statistics 20 software (SPSS Inc., Chicago, IL, USA), with a significance level of 0.05 (*p* < 0.05).

## 5. Conclusions 

In conclusion, glyceollins have the potential to be utilized in preventive approaches against neurodegenerative disorders as well as age-related memory deficits. Future studies on the cognition-enhancing effect of glyceollins in humans and their precise mode of action are warranted for their use as a food ingredient and for medical purposes.
